# Vector competence of Virginia mosquitoes for Zika and Cache Valley viruses

**DOI:** 10.1186/s13071-020-04042-0

**Published:** 2020-04-10

**Authors:** Kevin K. Chan, Albert J. Auguste, Carlyle C. Brewster, Sally L. Paulson

**Affiliations:** 1 Department of Entomology, Virginia Tech, Blacksburg, VA USA; 2 Plant and Environmental Sciences Department, Clemson University, Clemson, SC USA

**Keywords:** Zika virus, Cache Valley virus, Vector competence, *Aedes japonicus*, *Aedes albopictus*, *Aedes triseriatus*, *Aedes aegypti*, *Culex pipiens*, *Culex restuans*, Infection barriers

## Abstract

**Background:**

Vector-borne diseases are a major public health concern and cause significant morbidity and mortality. Zika virus (ZIKV) is the etiologic agent of a massive outbreak in the Americas that originated in Brazil in 2015 and shows a strong association with congenital ZIKV syndrome in newborns. Cache Valley virus (CVV) is a bunyavirus that causes mild to severe illness in humans and ruminants. In this study, we investigated the vector competence of Virginia mosquitoes for ZIKV and CVV to explore their abilities to contribute to potential outbreaks.

**Methods:**

To determine vector competence, mosquitoes were fed a blood meal comprised of defibrinated sheep blood and virus. The presence of midgut or salivary gland barriers to ZIKV infection were determined by intrathoracic inoculation *vs* oral infection. After 14-days post-exposure, individual mosquitoes were separated into bodies, legs and wings, and saliva expectorant. Virus presence was detected by plaque assay to determine midgut infection, dissemination, and transmission rates.

**Results:**

Transmission rates for *Ae. albopictus* orally infected (24%) and intrathoracically inoculated (63%) with ZIKV was similar to *Ae. aegypti* (48% and 71%, respectively). Transmission rates of ZIKV in *Ae. japonicus* were low, and showed evidence of a midgut infection barrier demonstrated by low midgut infection and dissemination rates from oral infection (3%), but increased transmission rates after intrathoracic inoculation (19%). *Aedes triseriatus* was unable to transmit ZIKV following oral infection or intrathoracic inoculation. CVV transmission was dose-dependent where mosquitoes fed high titer (ht) virus blood meals developed higher rates of midgut infection, dissemination, and transmission compared to low titer (lt) virus blood meals. CVV was detected in the saliva of *Ae. albopictus* (ht: 68%, lt: 24%), *Ae. triseriatus* (ht: 52%, lt: 7%), *Ae. japonicus* (ht: 22%, lt: 0%) and *Ae. aegypti* (ht: 10%; lt: 7%). *Culex pipiens* and *Cx. restuans* were not competent for ZIKV or CVV.

**Conclusions:**

This laboratory transmission study provided further understanding of potential ZIKV and CVV transmission cycles with *Aedes* mosquitoes from Virginia. The ability for these mosquitoes to transmit ZIKV and CVV make them a public health concern and suggest targeted control programs by mosquito and vector abatement districts.
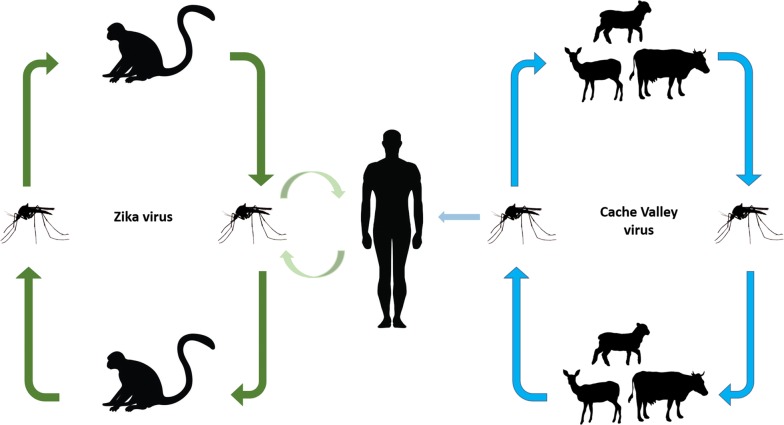

## Background

Vector-borne pathogens are a major public health concern and cause significant morbidity and mortality globally. In recent years, vector-borne pathogens have appeared in new regions, even as endemic diseases have increased in incidence. Human travel and trade are often responsible for the introduction of invasive pathogens but ecological factors such as climate and presence of competent vectors will determine whether the pathogen becomes established. For example, since its introduction in 1999, West Nile virus (WNV) (family *Flaviviridae*, genus *Flavivirus)* is now the leading cause of vector-borne encephalitis in the USA [1]. Also impacting vector-borne disease emergence are invasive mosquitoes that may alter the transmission cycles of pathogens, whether native or introduced [2]. *Aedes albopictus* and *Ae. japonicus* are two of the most invasive mosquito species worldwide [3] and both have been known to function as competent vectors for several enzootic mosquito-borne viruses in the USA [4, 5].

Zika virus (ZIKV) (family *Flaviviridae*, genus *Flavivirus*) is an arthropod-borne virus (arbovirus) of humans and has been linked to congenital malformations and microcephaly in developing fetuses, and Guillain-Barré syndrome in adults [6]. Since its introduction to Brazil in 2015, ZIKV has spread into many new areas within the Americas [7]. ZIKV is transmitted primarily by urban and sylvatic *Aedes* mosquitoes, with *Ae. aegypti* serving as the main vector for human infection outside of Africa [8–10]. This emerging mosquito-borne virus has caused epidemics throughout Africa, Asia, the Pacific Islands and the Americas [11, 12]. Due to the lack of knowledge of ZIKV replication in North American mosquitoes, experimental vector competence studies are necessary to better understand the potential transmission of ZIKV by additional species. Recent studies have shown that some *Aedes*, *Culex* and *Coquillettidia* mosquitoes from temperate regions of North America were not competent for ZIKV [13, 14], but this is a small representation of the species and strain diversity of mosquitoes that are found in the USA.

Cache Valley virus (CVV) (family *Peribunyaviridae*, genus *Orthobunyavirus*) is a neuroinvasive arbovirus that is also spread by mosquitoes. Although CVV infection typically causes mild symptoms in humans, fever, meningitis, and encephalitis have been reported [15]. The symptoms of CVV infection are more severe in ruminants, such as sheep or cattle, and include stillbirths, congenital malformations, spontaneous abortions, and death [16]. CVV has a widespread distribution in North America and has been isolated from many species of mosquitoes including *Ae. albopictus* and *Ae. japonicus* [17–20]. The principal vector is unknown, but vector competence studies and field isolations have shown that *Anopheles quadrimaculatus* and *An. punctipennis* may play a significant role in the natural transmission cycle [21, 22]. Laboratory transmission studies have also shown that *Cx. tarsalis*, *Ae. taeniorhynchus*, *Ae. sollicitans* and *Cq. perturbans* are competent vectors of CVV [22–24].

*Aedes aegypti* and *Ae. albopictus* are the most important mosquito species responsible for virus transmission to humans in urban environments. Both species are competent vectors for ZIKV, dengue virus (DENV) and yellow fever virus (YFV) [25–27]. The Asian rock pool mosquito, *Ae. japonicus*, is a relatively new invasive species that can be found in subtropical and temperate regions of the USA. Although *Ae. japonicus* is not an aggressive human biter, blood meal analysis from field collected mosquitoes have shown high incidences of human blood consumption [28]. Laboratory transmission studies show that *Ae. japonicus* is a competent vector of WNV, La Crosse virus (LACV), Eastern equine encephalitis virus (EEE) and St. Louis encephalitis virus (SLEV) [29–32]. *Aedes triseriatus*, the principal vector of LACV, is found extensively throughout eastern USA and parts of Central America [33]. Under laboratory conditions, *Ae. triseriatus* is a competent vector for WNV, DENV, YFV, EEE and SLE [34]. WNV has been isolated from *Culex pipiens* and *Cx. restuans* and both species have been shown to be competent vectors of the virus [35, 36]. Laboratory transmission studies have found that *Cx. pipiens* is refractory to CVV and ZIKV infections [13, 23, 25, 37, 38].

Between 2015 and 2018, there were more than 5000 imported ZIKV cases in the USA, with over 100 cases in Virginia [39]. Within the continental USA, reports of local transmission by mosquito vectors have occurred in Florida and Texas [40–42]. There have been no human CVV cases reported in Virginia, but the virus has been isolated from field-collected *Ae. japonicus* within the state [20]. Although CVV has been detected in field mosquitoes, only a few transmission studies have been conducted to determine potential vectors for the virus. With the wide distribution of *Ae. albopictus*, *Ae. japonicus*, *Ae. triseriatus*, *Cx. pipiens* and *Cx. restuans* throughout Virginia [43], it is crucial to determine the vector competence of these local mosquito strains. In this study, we investigated the vector competence of Virginia mosquitoes common in urban and suburban environments for ZIKV and CVV to explore their abilities to contribute to potential outbreaks and help inform local mosquito control strategies.

## Methods

### Mosquito collection and rearing

All eggs were derived from female mosquitoes collected using gravid traps in forested areas around Blacksburg, VA. After laying eggs, adult mosquitoes were tested for arboviruses by Vero cell plaque assay to ensure the absence of virus in the F1 progeny. A laboratory strain of *Ae. aegypti* from Vero Beach, FL, was used as our reference vector species and was subsequently tested for CVV vector competence. Mosquitoes were reared in environmental chamber conditions set at 24 °C with 75% RH and 16L:8D photoperiod using methods by Jackson et al. [44] to ensure consistent adult size.

### Cells and virus

African green monkey kidney (Vero) cells (American Type Culture Collection, Manassas, VA, USA) were cultured in Dulbecco’s Modified Eagle’s Medium (DMEM, Corning, Corning, NY, USA) with 10% fetal bovine serum (FBS), 100 U/ml of penicillin and 100 μg/ml of streptomycin, and maintained at 37 °C with 5% CO_2_.

The Asian lineage of ZIKV, PRVABC59 (GenBank: KU501215) and CVV strain 4B (GenBank: KX583998) was used in this study. PRVABC59 was isolated from the serum sample of a patient traveling from Puerto Rico in 2015. CVV4B was isolated from field-caught *Ae. japonicus* during a 2015 field study in Blacksburg, VA. Both viruses were maintained through passage on Vero cells and stored at − 80 °C. Infected blood meals consisted of 1 ml of virus mixed with 9 ml of defribinated sheep blood (Colorado Serum Company, Denver, CO, USA).

### Mosquito infection

For oral infection, 1-week-old female mosquitoes were starved 24 h before blood-feeding and provided cotton balls soaked with deionized water. Approximately 40–50 female mosquitoes were placed into 1-gallon cages covered with a mesh screen top. The mosquitoes were offered infected blood meals contained in a glass water-jacketed membrane feeder attached to a circulating 37 °C water bath. Pig intestine sausage casing was used as the membrane. After a 2-h feeding period, fully engorged females were anesthetized on ice and transferred to a new 1-liter cage. A 0.5 ml sample of the infected blood was removed after the feeding period and stored at − 80 °C for later virus titer. Parenteral infection was done by intrathoracic inoculation of week-old females that had never taken a blood meal with 0.2 µl of virus [45]. Table 1 shows the titers of virus infected blood meals and virus inoculum. Infected mosquitoes were maintained at 24 °C with 75% RH and 16L: 8D photoperiod and provided 10% sucrose solution for sustenance.

**Table 1:** ZIKV and CVV blood meal titers

Species	CVV high blood meal titer (pfu/ml)	CVV low blood meal titer (pfu/ml)	ZIKV blood meal titer (pfu/ml)	ZIKV intrathoracic inoculation titer (pfu/ml)
*Aedes albopictus*	5.25 × 10^6^	2.90 × 10^3^	3.00 × 10^6^	5.25 × 10^4^
*Aedes aegypti*	1.98 × 10^7^	6.28 × 10^3^	6.50 × 10^6^	5.25 × 10^4^
*Aedes japonicus*	1.99 × 10^6^	4.60 × 10^3^	3.72 × 10^7^	7.50 × 10^4^
*Aedes triseriatus*	2.99 × 10^6^	1.40 × 10^3^	4.50 × 10^7^	1.80 × 10^5^

### Saliva extraction

After 14-DPE, female mosquitoes were removed from cages and immobilized by chilling on ice. Saliva was extracted by inserting the proboscis into a capillary tube filled with a 1:1 mixture of 10% sucrose and fetal bovine serum (FBS) [46]. The mosquitoes were given 30 min to feed and salivate. The saliva, legs and wings and body were placed into separate microcentrifuge tubes with DMEM and stored at − 80 °C until virus testing.

### Virus detection

Mosquito bodies, leg and wing and saliva samples were homogenized with metal pellets in 2 ml of Vero media on a vortex mixer and then clarified by centrifugation at 1500× *g* for two min. Supernatants were tested for infection using Vero cell plaque assay following the methods of Barker et al. [47]. If virus was recovered from the body but not the legs and wings, the mosquito was classified as having a non-disseminated infection; if virus was detected in the wings and legs, the mosquito was classified as having a disseminated infection; mosquitoes with virus in saliva were classified as transmitting. Infection rate was determined as the percentage of the orally infected mosquitoes positive for virus in the body. Dissemination rate was determined as the percentage of orally infected mosquitoes that were positive for virus in the legs and wings, regardless of infection status. Transmission rate was determined as the percentage of orally infected mosquitoes that had virus in the salivary expectorant, regardless of infection status. Infectious blood meals were thawed at room temperature, diluted with a series of 10-fold serial dilutions and tested for virus concentration using plaque assay.

### Statistical analysis

A Chi-square test was used to compare mean infection, dissemination and transmission rates among mosquito species followed by Fisher’s exact tests for pairwise comparisons [48]. GraphPad Prism 6.0 (La Jolla, CA, USA) was used for all statistical analysis. All statistical analyses were carried out at a significance level of α = 0.05.

## Results

### Vector competence for ZIKV following oral infection

**Fig. 1:**
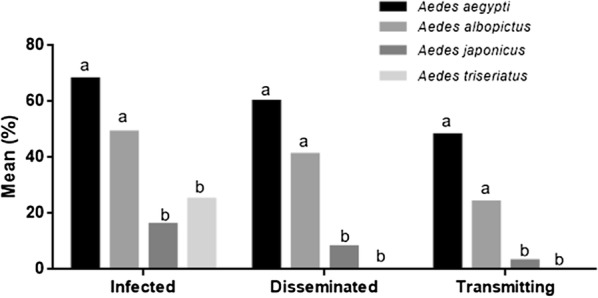
Vector competence for ZIKV PRVABC59. *Aedes albopictus* (*n* = 37), *Ae. triseriatus* (*n* = 28), *Ae. japonicus* (*n* = 73) and *Ae. aegypti* (*n* = 25) were provided infectious blood meals with an average titer of 2.57 × 10^7^ pfu/ml (range = 5.75 × 10^6^ to 7.5 × 10^7^ pfu/ml). After 14 days, mosquitoes were dissected and the number infected (% mosquitoes with virus in the body), disseminated (% mosquitoes with virus in legs and wings, independent of infection status) and transmitting (% mosquitoes with virus in saliva expectorant, independent of infection status) were determined by Vero cell plaque assay. Different letters denote significance by two-tailed Fischer’s exact test and presented as mean % infected, disseminated and transmitting, α = 0.05

There was a significant difference among infection (*χ*^2^ = 58.73, *df* = 5, *P* < 0.05), dissemination (*χ*^2^ = 71.21, *df* = 5, *P* < 0.05) and transmission (*χ*^2^ = 60.17, *df* = 5, *P* < 0.05) rates for *Ae. aegypti*, *Ae. albopictus*, *Ae. japonicus* and *Ae. triseriatus* after oral infection with ZIKV (Fig. 1). Rates for infection, dissemination and transmission were highest for *Ae. aegypti* (68%, 60% and 48%, respectively) and *Ae. albopictus* (49%, 41% and 24%, respectively). *Aedes japonicus* rates of infection, dissemination and transmission (20%, 9% and 3%) were significantly lower than *Ae. aegypti* (Fisher’s exact test, *P* < 0.0001, OR: 0.9257, 95% CI: 0.0326–0.2629; *P* < 0.0001, OR: 0.05970, 95% CI: 0.0187–0.1899; *P* < 0.0001, OR: 0.03052, 95% CI: 0.0061–0.1527) and *Ae. albopictus* (Fisher’s exact test, *P* < 0.0006, OR: 0.2077, 95% CI: 0.0849–0.5076; *P* = 0.0002, OR: 0.1313, 95% CI: 0.0454–0.3800; *P* = 0.0008, OR: 0.08764, 95% CI: 0.0178–0.4314) (Fig. 1). Although 25% of *Ae. triseriatus* became infected after imbibing an infectious blood meal, there was no dissemination or transmission of the virus. Neither *Cx. pipiens* nor *Cx. restuans* were infected after oral exposure to ZIKV (Table 2).

**Table 2:** Vector competence of ZIKV and CVV in *Culex pipiens* and *Cx. restuans*

Mosquito species	Sample size (*n*)	Mean titer (pfu/ml)	Mean non-disseminated infection (%)	Mean disseminated infection (%)	Mean transmitting (%)
ZIKV					
*Cx. pipiens*	30	3.00 × 10^7^	0	0	0
*Cx. restuans*	28	5.25 × 10^6^	0	0	0
CVV					
*Cx. pipiens*	67	1.12 × 10^8^	1	1	0
*Cx. restuans*	30	7.70 × 10^7^	0	0	0

### Transmission of ZIKV following parenteral infection

Parenteral infection by intrathoracic inoculation resulted in significantly higher rates of transmission compared to oral infection in *Ae. albopictus* (63% parenteral *vs* 24% oral) (Fisher’s exact test, *P* = 0.0080, OR: 0.1875, 95% CI: 0.0566–0.6209) and *Ae. japonicus* (19% parenteral *vs* 3% oral) (Fisher’s exact test, *P* = 0.0212, OR: 0.1197, 95% CI: 0.0202–0.7088) (Table 3). Mode of infection had no effect on transmission by *Ae. aegypti* (71% parenteral *vs* 48% oral) (Fisher’s exact test, *P* = 0.1395, OR: 0.3693, 95% CI: 0.1079–1.2630). No virus was detected in the saliva of *Ae. triseriatus* from either orally or parenterally infected groups (Table 3).

**Table 3:** Transmission rate of *Aedes japonicus* and *Ae. triseriatus* intrathoracically inoculated with ZIKV

Mosquito species	Infection method	Sample size (*n*)	Virus titer (pfu/ml)	Transmission (%)
*Ae. albopictus*	Intrathoracic	19	5.25 × 10^4^	63^a^
	Oral	37	3.00 × 10^6^	24^b^
*Ae. aegypti*	Intrathoracic	21	5.25 × 10^4^	71^a^
	Oral	25	6.50 × 10^6^	48^a^
*Ae. japonicus*	Intrathoracic	21	7.50 × 10^4^	19^a^
	Oral	73	3.72 × 10^7^	3^b^
*Ae. triseriatus*	Intrathoracic	23	1.80 × 10^5^	0^a^
	Oral	28	4.50 × 10^7^	0^a^

*Notes*: Differing letters denote significance of transmission rates of the same species after oral or intrathoracic infection by two-tailed Fischer’s exact test, α = 0.05

### Vector competence to CVV

**Fig. 2:**
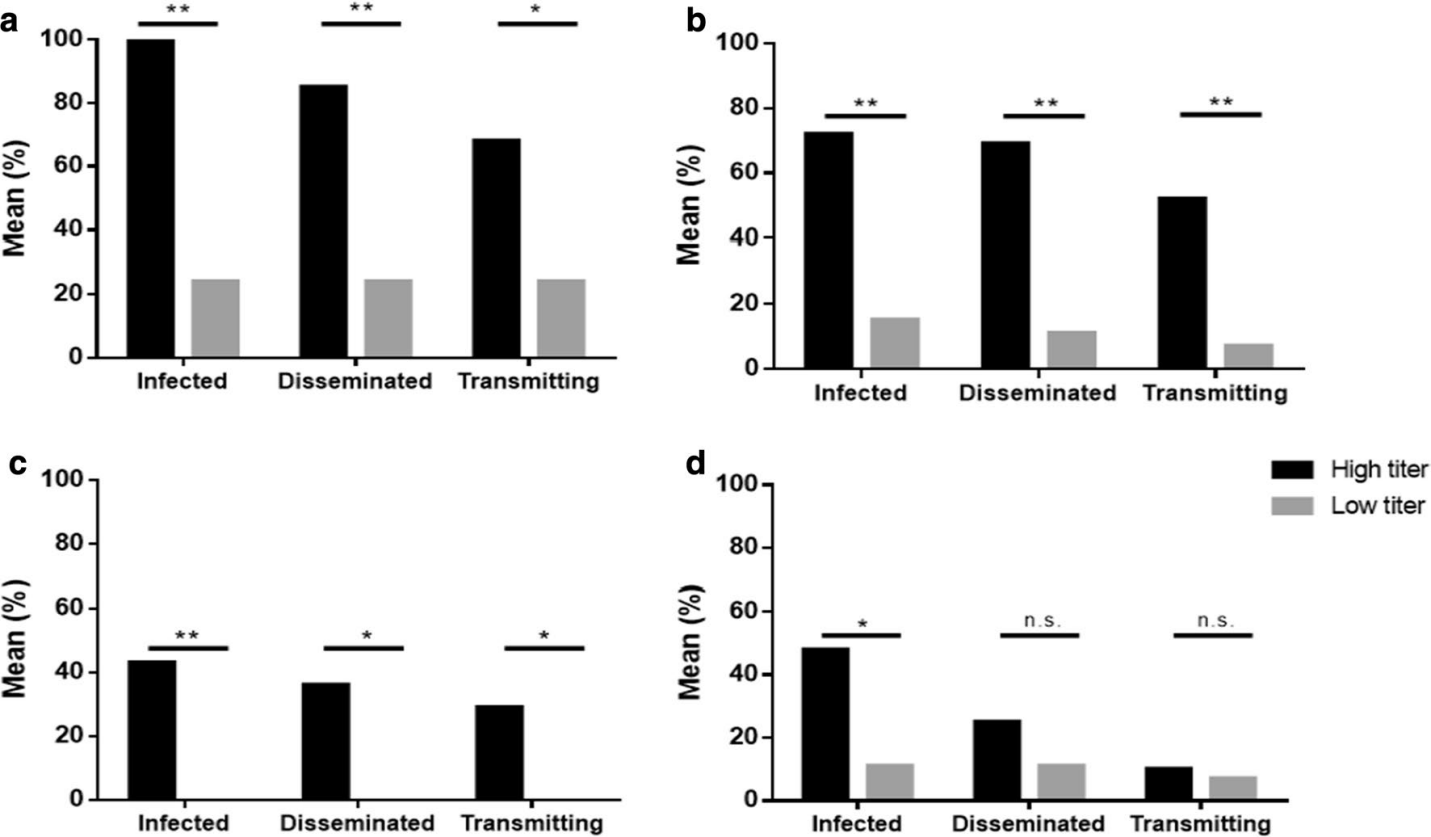
Vector competence for CVV with high *versus* low titer blood meals. Mosquitoes were provided low titer (lt) (1.2 × 10^3^ to 4.6 × 10^3^ pfu/ml) or high titer (ht) (1.6 × 10^5^ to 5.5 × 10^7^ pfu/ml) infectious blood meals. After 14 days, the mosquitoes were dissected and the number infected (% mosquitoes with virus in the body), disseminated (% mosquitoes with virus in legs and wings, independent of infections status) and transmitting (% mosquitoes with virus in saliva expectorant, independent of infection statues) were determined using Vero cell plaque assay. **a***Aedes albopictus* (lt: *n =* 21; ht: *n* = 34). **b***Aedes triseriatus* (lt: *n* = 55; ht: *n* = 29). **c***Aedes japonicus* (lt: *n* = 21; ht: *n* = 74). **d***Aedes aegypti* (lt: *n* = 44; ht: *n* = 52). Data are presented as mean % infected, disseminated and transmitting. *P < 0.01, **P < 0.001 and ns, not significant by two-tailed Fischer’s exact test

When fed a high titer (ht) virus blood meal, *Ae. albopictus* and *Ae. triseriatus* showed significantly higher rates of infection (*χ*^2^ = 127.5, *df* = 5, *P* < 0.0001), dissemination (*χ*^2^ = 107.8, *df* = 5, *P* < 0.0001) and transmission (*χ*^2^ = 88.08, *df* = 5, *P* < 0.0001) than *Ae. japonicus* or *Ae. aegypti* (Table 4). However, when fed low titer (lt) blood meals, there were no differences among rates for any of the species (*χ*^2^ = 5.61, *df* = 3, *P* > 0.05) (Table 4). *Aedes albopictus* was the most susceptible to CVV oral infection (ht: 100%, lt: 24%) and had the highest rate of dissemination (ht: 85%, lt: 24%) and transmission (ht: 68%, lt: 24%). *Aedes triseriatus* was also susceptible to CVV infection (ht: 72%, lt: 15%), dissemination (ht: 69%, lt: 11%) and transmission (ht: 52%, lt: 7%). None of the *Ae. japonicus* fed a low titer blood meal developed midgut infections. For *Ae. aegypti*¸ CVV was able to establish midgut infections (ht: 48%, lt: 11%), cause a disseminated infection (ht: 25%, lt: 11%) and transmit virus (ht: 10%; lt:7%). Figure 2 shows significant differences in infection, dissemination and transmission between high and low titer blood meals for *Ae. albopictus*, *Ae. triseriatus* and *Ae. japonicus*. Virus titer resulted in significant differences in infection for *Ae. aegypti* but not dissemination or transmission rates. Neither of the *Culex* species was able to transmit CVV. No *Cx. restuans* became infected and only one *Cx. pipiens* was positive for infection and dissemination (Table 2).

**Table 4:** Vector competence for CVV 4B by *Aedes albopictus*, *Ae. triseriatus*, *Ae. japonicus* and *Ae. aegypti* presented as mean % infected, disseminated and transmitting

Species	Sample size (*n*)	Mean blood meal titer (pfu/ml)	% infected	% disseminated	% transmitting
High titer					
* Ae. albopictus*	34	5.25 × 10^6^	100^a^	85^a^	68^a^
* Ae. triseriatus*	29	2.99 × 10^6^	72^a^	69^a^	52^a^
* Ae. japonicus*	74	1.99 × 10^6^	41^b^	38^b^	28^b^
* Ae. aegypti*	52	1.98 × 10^7^	48^a,b^	25^b^	10^b^
Low titer					
* Ae. albopictus*	21	2.90 × 10^3^	24^a^	24^a^	24^a^
* Ae. triseriatus*	55	1.40 × 10^3^	15^a^	11^a^	7^a^
*Ae. japonicus*	21	4.60 × 10^3^	0^a^	0^a^	0^a^
*Ae. aegypti*	44	6.28 × 10^3^	11^a^	11^a^	7^a^

*Notes*: Different letters denote significance between different species within respective categories of high or low titer blood meals by two-tailed Fischer’s exact test, α = 0.05

## Discussion

Assessing the vector competence of local mosquitoes for imported and emerging viruses is critical for public health officials to anticipate patterns of arbovirus transmission, determine the relative roles of the different species for virus amplification and spread, and to select appropriate control responses. This study aimed to determine the risk of local ZIKV transmission and the emergence potential of CVV in Virginia by evaluating the vector competence of the most common mosquito species found in urban and suburban habitats.

A meta-analysis by McKenzie et al. [49] suggested that the vector competence of *Ae. albopictus* for Zika virus varied among geographically disparate populations. We found that the vector competence of a Virginia strain of *Ae. albopictus* was equivalent to that of a Florida strain of *Ae. aegypti*. *Culex* mosquitoes were found to be refractory to ZIKV infection. Other studies have also observed similar results, suggesting that it is unlikely this group plays a role in ZIKV transmission [13, 37, 50, 51]. We also found that *Ae. japonicus* from Virginia was capable of transmitting ZIKV, but at a much lower rate compared to *Ae. aegypti* and *Ae. albopictus*. A study by Aliota et al. [13] showed that laboratory strains of *Ae. triseriatus* were able to become infected with ZIKV PRVABC59, the same strain that we used, but no dissemination or transmission resulted. Our study showed similar results working with an F1 generation of field-caught *Ae. triseriatus* where only midgut infections resulted from oral exposure.

Upon ingesting an infectious blood meal, the virus must surmount several tissue barriers associated with the midgut and salivary glands [52]. We assessed the presence of tissue barriers by intrathoracic inoculation of ZIKV. Injecting virus directly into the hemolymph bypasses the midgut and permits the virus to reach and infect the salivary glands. We detected infectious virus in salivary expectorant of *Ae. japonicus*, but not *Ae. triseriatus,* which indicated the presence of salivary gland barriers. Although transmission for intrathoracically inoculated *Ae. japonicus* was significantly higher than orally infected mosquitoes, the rates were low. The low midgut infection and transmission rates lead us to believe that there are potential midgut and salivary gland barriers that limit *Ae. japonicus* and prevent *Ae. triseriatus* from ZIKV transmission. Although *Ae. albopictus* was capable of ZIKV transmission after oral infection, intrathoracic inoculation significantly increased its transmission rates, indicating the presence of a midgut barrier. Studies of virus and vector systems have shown that these barriers play an important role during the extrinsic incubation period and may limit the ability of the virus to infect the mosquito for successful transmission [53, 54]. In addition, gut microbiota and immune pathways may also be involved when the virus enters the midgut [55–57]. It has been hypothesized that midgut and salivary gland barriers are responsible for the geographical variation in vector competence seen in *Ae. aegypti* and *Ae. albopictus* for ZIKV [58–60].

Although *Ae. japonicus* was capable of ZIKV transmission, it is not an aggressive human biter and predominantly inhabits forested areas, which limits its role in ZIKV transmission. Surprisingly, *Ae. triseriatus*, *Cx, pipiens* and *Cx. restuans* were not competent for ZIKV even though they are competent vectors of other flaviviruses, such as WNV or SLEV [61–63]. *Aedes albopictus*, on the other hand, was highly competent for ZIKV and exhibits aggressive, anthropophilic behaviour. The likelihood for this species to contribute to ZIKV transmission in Virginia is much higher compared to other *Aedes* mosquitoes in this region.

This study also found that *Ae. albopictus*, *Ae. triseriatus*, *Ae. japonicus* and *Ae. aegypti* were susceptible to CVV infection and capable of virus transmission. The combination of high vector competence, previous isolations from the field, and anthropophilic behavior suggests that *Ae. albopictus* could play a major role in CVV transmission in endemic areas [64, 65]. *Aedes triseriatus* and *Ae. japonicus* were also competent for CVV and blood meal analysis has shown that all three species feed on deer, the primary amplifying vertebrate host for CVV [28, 66–69]. We used high and low CVV blood meal titers that bracketed the range of titers found in experimentally infected deer [22] and showed that *Ae. albopictus*, *Ae. triseriatus* and *Ae. aegypti* were susceptible to CVV infection and subsequently transmitted virus even when exposed to low titer blood meals. There is currently no evidence of field isolation of the virus from *Ae. aegypti*, but the distribution of CVV includes the southern USA where *Ae. aegypti* is commonly found [18]. Even though CVV has been isolated from wild *Ae. japonicus* [20], and this species has been shown to feed on deer [28], it is unclear if it serves as a major vector in enzootic or local transmission of the virus. We also tested vector competence of field-caught *Cx. pipiens* and *Cx. restuans* for CVV, and found no evidence of transmission by either species. The existence of a dose-dependent infection or escape barrier can determine how certain mosquito species and strains are refractory to infection. Studies looking at dose-dependent interactions between mosquito vectors and the virus typically find that high titers result in greater midgut infection and transmission potential while low titers result in low midgut infection and transmission rates [70–73]. The dose-dependent tissue barriers are often associated with midgut escape barriers or RNA interference (RNAi) pathways [56, 72], while incompatibility between the virus and cells of the midgut or salivary glands are dose-independent barriers [52, 53]. With laboratory evidence of low titer vector competency and abundant distribution throughout North America, *Ae. aegypti*, *Ae. albopictus*, *Ae. japonicus* and *Ae. triseriatus* could play major roles in CVV transmission.

Outbreaks of mosquito-borne diseases can have large economic and devastating impacts on human and animal health. Experimental vector competence studies allow us to understand the potential for a mosquito species to contribute to an outbreak and facilitate more targeted surveillance and control. Due to the wide variability of mosquito and virus infectivity, it is not possible to make the assumption that studies involving vectors from different geographical locations will have similar competencies. Therefore, it is not appropriate to extrapolate results from other studies for a single conclusion. Several studies clearly show considerable variability in the susceptibility of the same vector species for viral infection for DENV [74, 75], CHIKV [76] and ZIKV [59, 77]. In addition, when arboviruses are detected in field-caught mosquitoes, we cannot assume that it is competent and able to transmit the virus. The mosquito may have an undigested blood meal that was recently taken from an infected host, which can yield a false positive. Laboratory vector competence studies allow us to determine if a mosquito species is capable of transmitting the virus. When conducting laboratory vector competence studies, it is important to consider laboratory-reared *versus* field-caught mosquitoes. For example, some vector competence studies have shown that laboratory-reared *Cx. quinquefasciatus* is able to become infected and transmit ZIKV [78, 79], while studies using field populations were not able to transmit the virus [38].

There are many knowledge gaps in CVV dynamics, especially our understanding of its natural cycle of competent vectors and susceptible amplifying hosts. In addition, CVV infections are often misdiagnosed for other flu-like illness, which presents itself as a challenge for accurate reporting to local or state health departments. In contrast, the ZIKV outbreak in 2015 sparked high demand for all areas of research to understand and control the virus. Although cases have dropped significantly in the USA, ZIKV is still present in parts of Africa, Asia and South America, and may remain indefinitely [11, 80].

## Conclusions

Our studies show that a species that has not been tested for ZIKV vector competency, *Ae. japonicus*, was able to transmit the virus, but at a low rate. *Aedes japonicus*, however, was competent for CVV transmission. *Aedes albopictus*, the most widespread anthropophilic mosquito in Virginia, was competent for both ZIKV and CVV. *Aedes aegypti* was competent for both viruses, but its inability to overwinter in colder climates reduces this species’ likelihood of ZIKV transmission in Virginia. With the abundance of highly competent mosquito species, there may be greater concern for increased CVV transmission in temperate regions of the USA.

## Data Availability

Data supporting the conclusions of this article are included within the article. The datasets used for the present study are available from the corresponding author upon request.

## Abbreviations

ZIKV: Zika virus
CVV: Cache Valley virus
WNV: West Nile virus
LACV: La Crosse virus
DENV: dengue virus
DPE: days post-exposure
lt: low titer
ht: high titer
